# Association of thyroid-stimulating hormone and lipid levels with suicide attempts among adolescents with major depressive disorder in China

**DOI:** 10.3389/fpsyt.2022.1031945

**Published:** 2023-01-17

**Authors:** Qing Zhang, Shuyi Zhao, Zhichun Liu, Bei Luo, Yingying Yang, Yudong Shi, Feng Geng, Lei Xia, Kai Zhang, Huanzhong Liu

**Affiliations:** ^1^Department of Psychiatry, School of Mental Health and Psychological Sciences, Anhui Medical University, Hefei, China; ^2^Department of Psychiatry, Chaohu Hospital of Anhui Medical University, Hefei, China; ^3^Anhui Psychiatric Center, Anhui Medical University, Hefei, China; ^4^Department of Psychiatry, Hefei Fourth People’s Hospital, Hefei, China

**Keywords:** depression, suicide attempts, thyroid-stimulating hormone, lipid levels, adolescents

## Abstract

**Objectives:**

Evidence suggested that thyroid-stimulating hormone (TSH) and lipid levels were associated with major depressive disorder (MDD). However, its role in suicide attempts in adolescents with MDD was unclear. This investigation was to probe into the relationship between TSH, lipid levels, and attempted suicide in adolescents with MDD.

**Methods:**

A total of 179 adolescents with MDD were included from January 2021 to January 2022. Socio-demographic data and clinical data were obtained through self-made questionnaires. TSH and lipid levels were analyzed by a recognized laboratory. The Epidemiological Studies Depression Scale (CES-D) was used to assess the severity of depression. Questions about whether there was a behavior of suicide attempts were completed through conversation interviews.

**Results:**

Results showed that the CES-D total score, TSH, and low-density lipoprotein cholesterol (LDL-C) of suicide attempters were significantly higher than those of non-suicide attempters. Ordinary family relationships were more likely to attempt suicide than good family relationships. The CES-D total score, ordinary family relationships, TSH and LDL-C were still significant in binary logistic regression, with an adjusted odds ratio (OR) of 1.04, 3.42, 5.14, and 1.76, respectively. The area under the receiver operating characteristic (ROC) curve showed that the area under the ROC curve (AUC) ranged from 0.60 to 0.74.

**Conclusion:**

Suicide attempts are common among adolescents with MDD and were associated with CES-D total score, ordinary family relationships, TSH, and LDL-C. Given the association between suicide attempts and TSH and LDL-C, the dynamic changes in TSH and LDL-C levels should be detected regularly.

## 1. Introduction

Major depressive disorder (MDD) is one of the most common mental disorders in adolescents and a major risk factor for suicidal behavior. A survey showed that MDD often begins in adolescence, seriously influences social functions, and increases the risk of recurrence in adulthood (1). Currently, suicide behavior in adolescents has increasingly become a public health concern. Surveys in the United States showed that the lifetime prevalence of suicide attempts in adolescents with MDD ranged from 5.6 to 8.9% (2, 3). Surveys of adolescents in China demonstrated that the prevalence of attempted suicide was 3.2–7.0% (4–6). However, the rate of suicide attempts in adolescents with MDD in China remains unknown. Prior studies have revealed some factors correlated with suicide attempts, including gender, family relationships, childhood trauma, and substance abuse (7–9). However, the precise mechanisms behind these influencing factors still need to be explored. At the same time, literature has shown that thyroid function and metabolic levels play an important role in suicide attempts.

Studies on the relationship between the hypothalamic-pituitary-thyroid (HPT) axis and depressive disorder have a long history. Literature has shown significantly higher thyroid-stimulating hormone (TSH) levels in people who have attempted suicide than in a non-suicidal group (10). In a recent study, researchers reported that compared with non-suicide attempters, patients who attempted suicide had statistically higher levels of TSH, anti-thyroglobulin, and anti-thyroid peroxidase (11). However, some inconsistent findings have been demonstrated. Data from one study suggested that those with higher TSH levels had fewer depressive symptoms and were less likely to commit suicide than those with lower TSH levels (12). The relationship between TSH and suicide attempts remains controversial. Furthermore, most studies in this field on the relationship between TSH and suicide attempts have only focused on the adult group, and few published investigations were found on adolescents. Therefore, it is still unknown whether suicide attempts are related to TSH levels among adolescents with MDD.

It is now well-established that depression shares similar pathways with cardiovascular disease (CVD), and patients with CVD generally showed a high prevalence of depression (13). Some researchers have explored the mechanisms of the relationship between cholesterol and suicidal behaviors. The cholesterol-serotonin hypothesis suggests that one of the functions of serotonin is to inhibit harmful impulses, and low membrane cholesterol levels reduce the number of serotonin receptors, which could contribute to a poorer suppression of aggressive behavior (14). However, mixed results were found. A meta-analysis of 65 epidemiological studies found that patients who were suicidal had significantly lower serum total cholesterol (TC), low-density lipoprotein cholesterol (LDL-C), and triglycerides (TG) levels compared with non-suicidal patients. However, another study of depressed patients showed that suicide attempters had higher TC and lower high-density lipoprotein cholesterol (HDL-C) levels and indicated that suicide attempts were associated with higher TC and lower LDL-C levels (15). In addition, two studies demonstrated that there was no connection between lipid levels and attempted suicide (16, 17). Thus far, studies on suicide attempts and lipid levels in adolescent patients with MDD are scarce. The relationship between lipids levels and attempted suicide in adolescents with MDD is still unclear and needs further exploration.

Although a few studies have examined the associations between suicide attempts and TSH and lipid levels, there is a lack of evidence for such an association among adolescents with MDD. In this investigation, we analyzed the prevalence and factors related to suicide attempts among adolescents with MDD and determined whether suicide attempts were associated with serum TSH and lipid levels.

## 2. Materials and methods

### 2.1. Study design and participants

From January 2021 to January 2022, 204 eligible patients were enrolled in this study. Twenty patients (9.8%) refused to participate, and five (2.4%) provided incomplete questionnaires, leading to a response rate of 87.7%. We enrolled 179 adolescents with MDD from outpatients and inpatients in two affiliated hospitals of Anhui Medical University in Anhui province (Chaohu Hospital of Anhui Medical University, Hefei Fourth People’s Hospital, China). We assigned these 179 adolescent participants to an MDD group. All patients were informed that they had the right to decide, refuse, or withdraw without giving any reason at any time. Each patient who decided to engage in this study signed formal written consent forms. The researchers divided the patients with MDD into two groups based on whether the patients had attempted suicide. This study received permission from the Ethics Committee of Chaohu Hospital, affiliated with Anhui Medical University (202009-kyxm-04).

The inclusion criteria for patients with MDD were as follows: (1) patients diagnosed with MDD by two senior psychiatrists using the fifth edition of the Diagnostic and Statistical Manual of Mental Disorders (DSM-5); (2) aged 13–18 years with a good level of Mandarin language comprehension and Han ethnicity; each enrolled patient received a thorough medical somatic checkup, personal disease history evaluation. Patients were excluded if they met any of the following criteria: (1) severe organic illness; (2) current use of non-steroidal anti-inflammatory drugs or immunomodulators, such as interferons; (3) alcohol or drug dependence other than tobacco smoking; (4) a diagnosis of intellectual disability or other types of mental disorders; (5) diagnosis of with thyroid diseases or prior thyroid surgery in the past; (6) dyslipidemia.

### 2.2. Socio-demographic characteristics

Trained investigators gathered socio-demographic data, such as age, gender, education level, body mass index (BMI), and family relationships (good, ordinary, or bad). Some clinical features, such as age at onset (years), medication, illness course (months), and recruitment in different settings, were also recorded by investigators.

### 2.3. Clinical measures

The 20-item Center for Epidemiological Studies Depression Scale (CES-D) was utilized to screen for the severity of depression (18). Each item was divided into four levels, from 0 to 3, for frequency of depressive symptoms during the past week (0 = “not at all,” 1 = “a little,” 2 = “some,” and 3 = “a lot”). The 20 unweighted scores were added together (items 4, 8, 12, and 16 with opposite polarity) for the CES-D total score. The higher the total score, the more severe the depressive symptoms. The Cronbach’s α for the CES-D is 0.80, which shows high internal consistency (19).

Suicide attempts were defined as the deliberate acts of self-harm with at least some intent to die (20). Assessment of lifetime suicide attempts was measured by the question. All enrolled subjects were asked, “Have you tried to commit suicide so far?” This question was taken from a survey by the World Health Organization (21). If the MDD patients’ answer to suicide attempts was “yes,” the patients were considered suicide attempters; otherwise, they were not.

### 2.4. Blood samples

Fasting venous blood was taken from each patient between 6:00 and 8:00 a.m., and all the patients maintained an overnight fast. Blood (10 ml) was collected from each patient. Prior to the examinations, the plasma was separated into several refrigerated storage tubes and placed in a −80°C freezer. TSH, TG, TC, HDL-C, and LDL-C were measured. TSH was tested using a Cobas E 801 Electrochemiluminescence Immunoassay Analyzer (Roche Diagnostics, Shanghai, China). TG was detected using the TG_2 Reagents method. TC was detected using the Cholesterol_2 Reagents method. HDL-C was detected using the Direct HDL Cholesterol Reagents method. LDL-C was detected using the LDL Cholesterol Direct Reagents method. The blood was centrifuged (3,000 rpm for 5 min) within 1 hour of the assay. The normal ranges were 0.27–4.20 μIU/ml for TSH, 0–5.18 mmol/L for TC, 0–1.7 mmol/L for TG, 1.04–1.42 mmol/L for HDL-C, and 0–3.37 mmol/L for LDL-C.

### 2.5. Statistical analysis

Data from quantitative variables are presented as mean ± standard deviation. Categorical data are presented as frequency distributions. The Kolmogorov–Smirnov one-sample test normality of continuous variables; when normally distributed, we used the Student’s *t*-test to check differences between the two groups. If not normally distributed, the data were analyzed using the Mann–Whitney *U* test. The chi-squared test was used to compare categorical variables. Partial correlations controlling for age, gender, recruitment in different settings, and medication were used to analyze correlations between suicide attempts, TSH, LDL-C, CES-D total score, and family relationships. After controlling relevant covariates, a binary logistic regression was used to identify significant independent variables associated with suicide attempts in adolescents with MDD. The area under the receiver operating characteristic (ROC) curve, area under the ROC curve (AUC), was used to measure the performance of parameters in distinguishing between patients who had attempted suicide and those who had not (22). AUC values range from 0.5 to 1; an AUC of 0.5 indicate that the continuous variable cannot determine a difference, AUCs of 0.5–0.7 indicate moderate diagnostic effectiveness, and AUCs > 0.8 indicate good diagnostic ability (23, 24). SPSS 23.0 statistical software was used for statistical analysis, and *P*-values less than 0.05 (two-tailed) were considered statistically significant. GraphPad Prism 9.0 was used for drawing the ROC curves.

## 3. Results

### 3.1. Socio-demographic and clinical factors between suicide attempts and non-suicide attempts groups in adolescents with MDD

Table 1 shows demographic and clinical features in the two patient groups. Of the 179 enrolled patients, 118 (65.92%) had attempted suicide, and 61 (34.08%) had not. There were no statistically significant differences in age, duration of illness, age at onset, BMI, education level, medication, recruitment in different settings, or the levels of TC, TG, and HDL-C (*P* > 0.05). Family relationships, CES-D total score, and levels of TSH and LDL-C were significantly different between the groups (all *P* < 0.05).

**TABLE 1:** Socio-demographics and clinical characteristics of adolescent patients between suicide attempters and non-suicide attempters.

Variable	All patients *N* = 179	Suicide attempters *N* = 118	Non-suicide attempters *N* = 61	/*Z*/χ^2^	*P*
Age (years)	15.40 ± 1.56	15.31 ± 1.62	15.59 ± 1.44	-1.01	0.312
Duration of illness (months)	22.42 ± 18.68	23.82 ± 19.66	19.70 ± 16.41	-1.30	0.193
Age at onset (years)	13.69 ± 1.96	13.50 ± 2.12	14.07 ± 1.57	-1.48	0.139
BMI (kg/m^2^)	20.99 ± 3.87	20.93 ± 3.89	21.09 ± 3.85	-0.22	0.820
Gender				3.86	0.050
Male [n (%)]	51 (28.50%)	28 (23.70%)	23 (37.70%)		
Female [n (%)]	128 (71.50%)	90 (76.30%)	38 (62.30%)		
Education level				1.29	0.255
Junior high school [n (%)]	75 (41.90%)	53 (44.90%)	22 (36.10%)		
High school [n (%)]	104 (58.10%)	65 (55.10%)	39 (63.90%)		
Family relationships				14.86	**0**.001**
Good [n (%)]	55 (30.70%)	25 (21.20%)	30 (49.20%)		
Ordinary [n (%)]	98 (54.70%)	73 (61.90%)	25 (41.00%)		
Bad [n (%)]	26 (14.50%)	20 (16.90%)	6 (9.80%)		
CES-D total score	37.41 ± 12.47	40.23 ± 10.91	31.97 ± 13.54	-3.86	**<0**.001***
**Hematological index**					
TSH (μIU/mL)	1.25 ± 0.24	1.29 ± 0.23	1.19 ± 0.26	2.48	**0**.014*
TG (mmol/L)	1.22 ± 0.71	1.24 ± 0.72	1.19 ± 0.70	-0.54	0.587
TC (mmol/L)	3.81 ± 0.98	3.91 ± 0.97	3.61 ± 0.96	-1.80	0.073
HDL-C (mmol/L)	1.15 ± 0.34	1.17 ± 0.33	1.11 ± 0.35	-0.69	0.490
LDL-C (mmol/L)	2.02 ± 0.76	2.11 ± 0.78	1.86 ± 0.70	2.11	**0**.036*
Medication				3.07	0.381
No treatment [n (%)]	75 (41.90%)	44 (37.30%)	31 (50.80%)		
SSRIs [n (%)]	97 (54.20%)	69 (58.50%)	28 (45.90%)		
SNRIs [n (%)]	4 (2.20%)	3 (2.50%)	1 (1.60%)		
Combination [n (%)^a^]	3 (1.70%)	2 (1.70%)	1 (1.60%)		
Recruitment in different settings				2.14	0.144
Inpatients [n (%)]	84 (46.90%)	60 (50.80%)	24 (39.30%)		
Outpatients [n (%)]	95 (53.10%)	58 (49.20%)	37 (60.70%)		

BMI, body mass index; CES-D, the Epidemiological Studies Depression Scale; TSH, thyroid-stimulating hormone; TG, triglycerides; TC, total cholesterol; HDL-C, high-density lipoprotein cholesterol; LDL-C, low-density lipoprotein cholesterol; SSRIs, selective serotonin reuptake inhibitors; SNRIs, serotonin-noradrenaline reuptake inhibitors. Bold values signify statistically significant results. *P < 0.05; **P < 0.01; and ***P < 0.001.

^a^Combination meant that SSRIs and SNRIs are used at the same time.

### 3.2. Partial correlations between suicide attempts, TSH, LDL-C, CES-D, and family relationships, controlling for age, gender, recruitment in different settings, and medication

Table 2 showed that suicide attempts were correlated with TSH levels, CES-D total score, and family relationships and not with LDL-C levels.

**TABLE 2:** Partial correlations between suicide attempts, TSH, LDL-C, CES-D total score, and family relationships, controlling for age, gender, recruitment in different settings, and medication.

	1	2	3	4	5
1-TSH	1				
2-LDL-C	**0.18** *	1			
3-CES-D total score	0.11 (N.S.)	−0.05 (N.S.)	1		
4-Suicide attempts	**0.19** *	0.13 (N.S.)	**0**.30***	1	
5- Family relationships	0.01 (N.S.)	−0.06 (N.S.)	**0**.40***	**0**.26**	1

CES-D, the Epidemiological Studies Depression Scale; TSH, thyroid-stimulating hormone; LDL-C, low-density lipoprotein cholesterol. Bold values signify statistically significant results. N.S. = Not Significant. *P < 0.05; **P < 0.01; and ***P < 0.001.

### 3.3. Related factors of suicide attempts in adolescents with MDD by binary logistic regression analysis and ROC analysis

**FIGURE 1:**
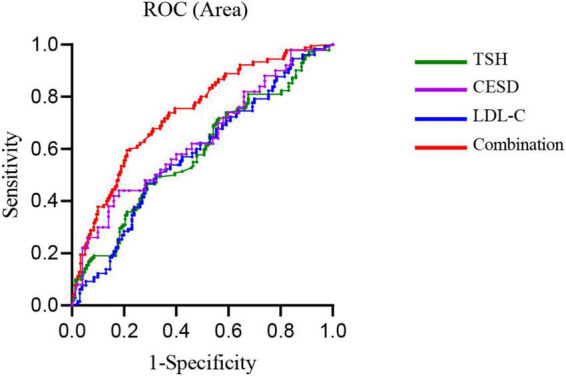
The receiver operating characteristic (ROC) curve. The ROC curve was used to determine the discriminatory capacity of parameters to distinguish between suicide attempters and non-suicide attempters. The area under the ROC curve (AUC) of thyroid-stimulating hormone (TSH), low-density lipoprotein cholesterol (LDL-C), Epidemiological Studies Depression Scale (CES-D) total score, and the combination of these three parameters were 0.60, 0.60, 0.68, and 0.74, respectively.

Demographic and clinical variables were adjusted by using binary logistic regression analysis. As shown in Table 3, TSH [odds ratio (OR) = 5.14, 95% confidence interval (CI) = 1.07–24.68, *P* = 0.04], LDL-C (OR = 1.76, 95% CI = 1.04–2.99, *P* = 0.04), ordinary family relationships (OR = 3.42, 95% CI = 1.50–7.80, *P* = 0.003), and CES-D total score (OR = 1.04, 95% CI = 1.01–1.08, *P* = 0.011). The AUCs of each related factor was as follows: CES-D, 0.68; TSH, 0.60; LDL-C, 0.60; the combination of CES-D total score, TSH, and LDL-C, 0.74. The ROC curves of each parameter are shown in Figure 1.

**TABLE 3:** Adjusted odds ratio (OR) for factors associated with suicide attempts and receiver operating characteristic (ROC) curve analysis results.

Parameters	Logistic regression (Adjusted^a^)		ROC analysis	
	OR (95% CI)	*P*	AUC (95% CI)	*P*
**Family relationships**	–	–	–	-
Good	Ref	Ref	–	-
Ordinary	3.42 (1.50–7.80)	**0.003** **	–	-
Bad	2.84 (0.84–9.62)	0.09	–	-
CES-D total score	1.04 (1.01–1.08)	**0.011** *	0.68 (0.59–0.76)	**<0**.001***
TSH	5.14 (1.07–24.68)	**0.04** *	0.60 (0.51–0.69)	**0**.03*
LDL-C	1.76 (1.04–2.99)	**0.04** *	0.60 (0.51−0.69)	**0**.03*
Combination^b^	–	–	0.74 (0.65–0.81)	**<0**.001***

CES-D, the Epidemiological Studies Depression Scale; TSH, thyroid-stimulating hormone; LDL-C, low-density lipoprotein cholesterol.

^a^Suicide attempts model was adjusted with age, gender, and medication.

^b^Combination of CES-D, TSH, and LDL-C. Bold values signify statistically significant results.

*P < 0.05; **P < 0.01; and ***P < 0.001.

## 4. Discussion

To the best of our knowledge, this was the first study to examine relationships between TSH, lipid levels, and suicide attempts in adolescents with MDD. We found the prevalence of suicide attempts in the patient sample was 65.92%, significantly higher than the previous data. A large sample of hospitalized adolescents from the United States found that the prevalence of attempted suicide was 38.6% (25). The results of our higher prevalence of suicide attempts supported the view that depression was closely associated with suicide behaviors (26). The ROC analysis indicated the association between CES-D total score, LDL-C, TSH, and suicide attempts. The combination of CES-D total score, TSH, and LDL-C had an AUC value of 0.74, suggesting moderate diagnostic effectiveness.

Earlier research found a relationship between lipid levels and suicide behaviors (27, 28). In the present investigation, adolescents with higher serum LDL-C levels were more likely to attempt suicide than those with lower LDL-C levels. These results were consistent with a recent cross-sectional study involving 580 patients with MDD aged 18–60 years, which found suicide attempts were associated with increased TC and LDL-C levels (29). Similarly, another survey showed that for every 0.26 mmol/L increase in the LDL-C level of women, the risk of suicide could increase by 10% (30). However, other studies demonstrated lower serum lipids levels correlated negatively with an increased risk of suicidal behaviors in patients with MDD (31, 32). The relationship between serum LDL-C levels and attempted suicide showed significant heterogeneity. The patient’s clinical conditions could potentially cause this discrepancy in the relationship between serum LDL-C levels and suicide attempts. Some patients in the present study had accepted pharmacological treatment, which could have influenced their blood lipid levels (33). Age, gender, diet, and improper fasting before sampling could affect measured blood lipid levels (34, 35).

The present study also found that suicide attempters had higher serum TSH levels than patients who did not attempt suicide, and the serum TSH levels were associated with the risk of suicide attempts. However, another study revealed TSH levels were significantly lower in those who attempted suicide than in those who did not (36). There are possible explanations for this discrepancy. First, a previous study found that thyrotropin-releasing hormone had a higher response to TSH in suicide attempters, stimulating TSH secretion (37). Second, a survey of the effect of long-term oral estradiol on the HPT axis in rats reported that TSH and total thyroxine levels increased in rats treated with estradiol orally. Moreover, it also revealed that oral estradiol stimulated thyrotropin-releasing hormone to induce the secretion of TSH (38). At the same time, a large sample study on the relationship between menopausal hormone therapy and suicide risk in middle-aged and older women showed that hormone therapy increased the risk of suicide attempts by 1.4-fold (39). Currently, the percentage of women is 76.30% (90/118) among suicide attempters, accounting for a large proportion. We inferred that the correlation between hormones and the HPT axis would be one of the reasons for the higher TSH levels in suicide attempters.

The results showed that ordinary family relationships and CES-D total score were related factors of attempted suicide among adolescents with MDD. Previous research has identified risk factors for suicide attempts similar to our findings (4, 40). Good family relationships and cohesion could make adolescents better control their emotions and reduce the conversion from suicidal ideation to suicide attempts (41).

The present study found that TSH and LDL-C levels were associated with suicide attempts. However, this study also had several limitations. First, some participants in this study were taking antidepressants that could have affected levels of blood measurements. Second, this was a cross-sectional study, and the causal associations between serum TSH and LDL-C levels and suicide attempts could not be established; hence, these findings should be validated using large longitudinal and prospective studies. Third, the determination of suicide attempt history was performed by an interview, which could have introduced recall bias and lacked structured suicide assessment tools. Fourth, smoking affects lipid levels, and information about smoking was not collected. Fifth, we used a self-reported scale rather than a comprehensive measure of depression, such as the Hamilton Rating Scale for Depression (HAM-D), to evaluate depression severity, which might introduce biases. Moreover, due to time constraints, patients were not asked detailed questions, such as the frequency, specific methods, or dates of suicide attempts, which resulted in the loss of information. Future work should improve on this study to further probe into the causes of attempted suicide among adolescents with MDD.

## 5. Conclusion

In summary, among adolescents with MDD, suicide attempters were more likely to have severe depressive symptoms and higher TSH and LDL-C levels. Considering the high prevalence of suicide attempts among adolescents with MDD and the association between suicide attempts, TSH, and LDL-C, regular detection of dynamic changes in TSH and LDL-C and improvements of the parent–child relationships should be undertaken.

## Data availability statement

The raw data supporting the conclusions of this article will be made available by the authors, without undue reservation.

## Ethics statement

The studies involving human participants were reviewed and approved by the Medical Ethics Committee of Chaohu Hospital Affiliated to Anhui Medical University. The Ethics number is 202009-kyxm-04. Written informed consent to participate in this study was provided by the participants’ legal guardian/next of kin.

## Author contributions

QZ, SZ, and ZL: writing—original draft. BL, YY, YS, FG, LX, KZ, and HL: reviewing and editing. All authors contributed to the article and approved the submitted version.
